# Late Onset of the Serological Response against the 18 kDa Small Heat Shock Protein of *Mycobacterium ulcerans* in Children

**DOI:** 10.1371/journal.pntd.0002904

**Published:** 2014-05-22

**Authors:** Katharina Röltgen, Martin W. Bratschi, Amanda Ross, Samuel Y. Aboagye, Kobina A. Ampah, Miriam Bolz, Arianna Andreoli, James Pritchard, Jacques C. Minyem, Djeunga Noumen, Eric Koka, Alphonse Um Boock, Dorothy Yeboah-Manu, Gerd Pluschke

**Affiliations:** 1 Swiss Tropical and Public Health Institute, Molecular Immunology, Basel, Switzerland; 2 University of Basel, Basel, Switzerland; 3 Noguchi Memorial Institute for Medical Research, University of Ghana, Legon, Ghana; 4 FAIRMED, Yaoundé, Cameron; 5 Bankim District Hospital, Bankim, Cameroon; Fondation Raoul Follereau, France

## Abstract

A previous survey for clinical cases of Buruli ulcer (BU) in the Mapé Basin of Cameroon suggested that, compared to older age groups, very young children may be less exposed to *Mycobacterium ulcerans*. Here we determined serum IgG titres against the 18 kDa small heat shock protein (shsp) of *M. ulcerans* in 875 individuals living in the BU endemic river basins of the Mapé in Cameroon and the Densu in Ghana. While none of the sera collected from children below the age of four contained significant amounts of 18 kDa shsp specific antibodies, the majority of sera had high IgG titres against the *Plasmodium falciparum* merozoite surface protein 1 (MSP-1). These data suggest that exposure to *M. ulcerans* increases at an age which coincides with the children moving further away from their homes and having more intense environmental contact, including exposure to water bodies at the periphery of their villages.

## Author Summary

Although *M. ulcerans,* the causative agent of Buruli ulcer (BU), was identified in 1948, its transmission pathways and environmental reservoirs remain poorly understood. The occurrence of *M. ulcerans* infections in endemic countries in West and Central Africa is highly focal and associated with stagnant and slow flowing water bodies. BU is often described as a disease mainly affecting children <15 years of age. However, taking the population age distribution into account, our recent longitudinal survey for BU in the Mapé Dam Region of Cameroon revealed that clinical cases of BU among children <5 years are relatively rare. In accordance with these findings, data of the present sero-epidemiological study indicate that children <4 years old are less exposed to *M. ulcerans* than older children. Sero-conversion is associated with age, which may be due to age-related changes in behavioural factors, such as a wider movement radius of older children, including more frequent contact with water bodies at the periphery of their villages.

## Introduction

It has been established that the chronic necrotizing skin disease BU is caused by the emerging pathogen *Mycobacterium ulcerans*, however the mode(s) of transmission and environmental reservoirs are still unknown. Comparative genetic studies have revealed that *M. ulcerans* has diverged from the fish pathogen *M. marinum*. Through the acquisition of a plasmid, *M. ulcerans* has gained the ability to produce a cytotoxic and immunosuppressive macrolide toxin, referred to as mycolactone [1], [2]. In addition to *M. ulcerans* strains isolated from human lesions, which belong either to the classical or to the ancestral lineage [3], other mycolactone-producing mycobacteria (MPM) have been identified as fish and frog pathogens and given diverse species names [4]–[7]. However, recent comparative genomic analyses have shown that all MPM are genetically closely related and can be divided into three principal ecovars of *M. ulcerans* [8]. Extensive pseudogene formation and genome downsizing of the human *M. ulcerans* pathogen are indicative for an adaptation to a more stable ecological niche. In African endemic settings both the physical environment and organisms such as amoeba, insects, fish and frogs have been proposed as possible environmental reservoirs of the pathogen [9]. Accordingly, direct inoculation of bacteria into the skin from an environmental reservoir, but also bites from insects, such as mosquitos or water bugs have been suggested as route of infection. While possums have been identified as an animal reservoir in BU endemic areas of Southern Australia [10], no mammalian reservoir has so far been detected in Africa. The distribution pattern of lesions is not indicative for a particular route of infection [11] and a genetic fingerprinting study of *M. ulcerans* isolates has revealed a highly focal transmission pattern, which excludes certain modes of transmission [12].

While it has long been generalized that in African BU endemic areas children below the age of 15 are most affected by the disease [13], population age-stratified data from our previous survey for BU in the Mapé Basin of Cameroon showed that children less than five years old were underrepresented among cases [11]. One explanation for this observation may be a lower degree of exposure of very young children to *M. ulcerans*. Sero-epidemiological studies in Ghana have shown that screening blood sera of local populations for the presence of IgG specific for the 18 kDa shsp of *M. ulcerans* represents a tool to monitor exposure of populations to *M. ulcerans* [14]. However, in these investigations study participants were older than five years of age. Since a proportion of study participants of all age groups tested positive, it is still not known at which age immune responses against *M. ulcerans* start to emerge and hence where and at which age exposure to the pathogen begins.

In the present sero-epidemiological study the potential association between age and exposure to *M. ulcerans* was investigated by determining anti-18 kDa shsp IgG titres in 875 individuals from BU endemic sites in the Densu River Basin of Ghana and the Mapé Basin of Cameroon. In these cross-sectional surveys we included more than 100 children less than five years old allowing us to estimate the age of sero-conversion, which may provide another cornerstone in the search for the mode of *M. ulcerans* transmission.

## Materials and Methods

### Ethics statement

Ethical clearance for the collection and testing of human blood samples from Ghana and Cameroon was obtained from the institutional review board of the Noguchi Memorial Institute for Medical Research (Federal-wide Assurance number FWA00001824) and the Cameroon National Ethics Committee (N°172/CNE/SE/201) as well as the Ethics Committee of Basel (EKBB, reference no. 53/11). Written informed consent was obtained from all individuals involved in the study. Parents or guardians provided written consent on behalf of children.

### Study design

We investigated the association between age and exposure to *M. ulcerans* by determining serum antibody titres against the 18 kDa shsp in individuals living in two different BU endemic areas.

In Cameroon, serum samples were collected from inhabitants of the village of Mbandji 2. This village is located in the Bankim Rural Health Area of the Bankim Health District, where we conducted a cross-sectional house-by-house survey for BU in early 2010, including the collection of data on the population age structure. These data and the subsequent identification of BU cases until June 2012 were published in our previous study [11]. In the present study we provide updated information based on a continued monitoring of new BU cases in this area until May 2013. The age-specific incidence rates were calculated using the ages of the BU cases identified between March 2010 and May 2013 and the population age distribution as collected in the house-by-house survey in the Bankim Health District.

Sera were collected in January 2011 from all inhabitants of Mbandji 2, who agreed to participate (395 individuals with a nearly equal gender distribution). Re-sampling of 80 blood donors from Mbandji 2 was carried out one year after the first blood collection to analyze stability of anti-18 kDa shsp serum IgG levels over time.

The second study site comprised villages within the Obom sub-district of the Ga-South district in Ghana. This sub-district is one of the major BU endemic communities along the Densu River Basin. The villages from which the sera were collected, have active transmission on-going as they have continuously reported cases for the past five years. Study participants included 96 laboratory confirmed BU patients (57 females and 39 males) as well as 4 age-, sex-, and home village-matched controls for each patient (384 control individuals).

**Figure 1:**
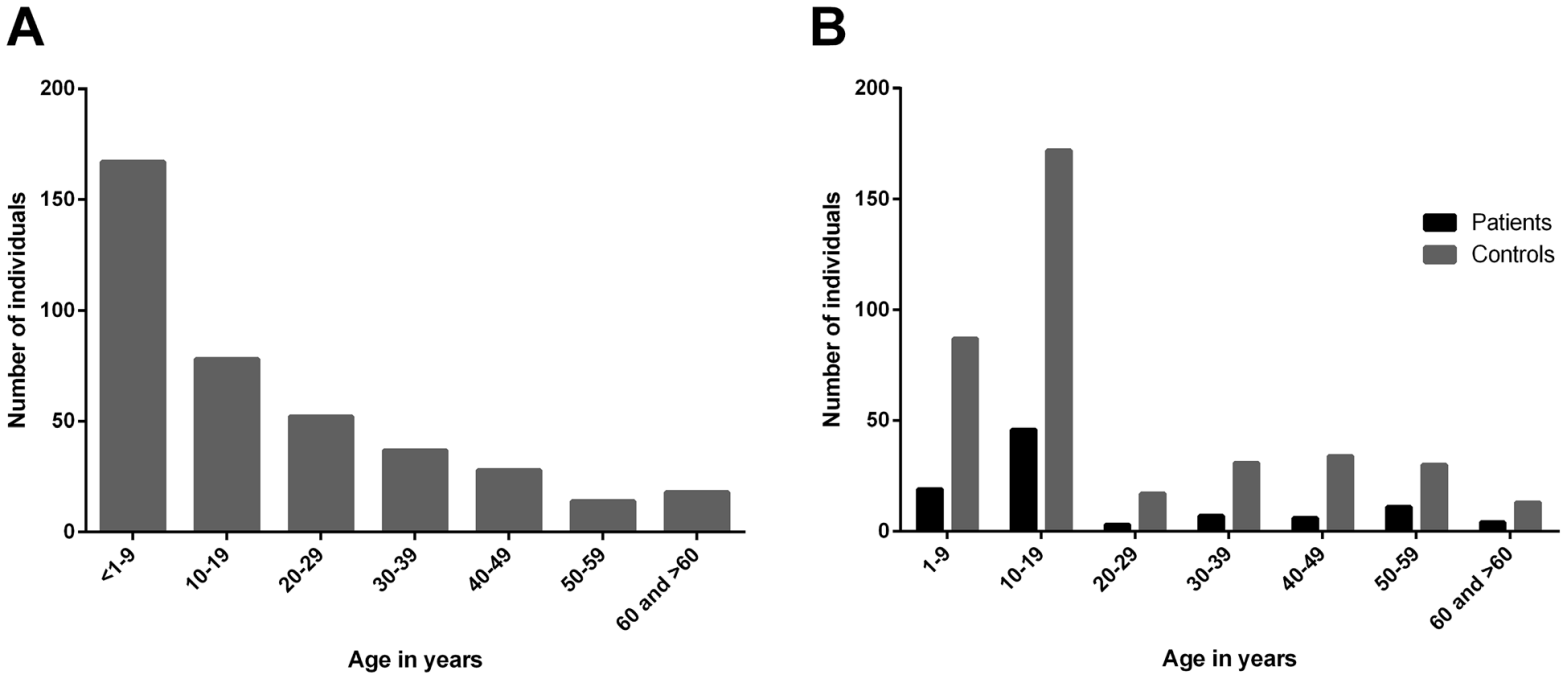
Age distribution of study participants. **A** In Cameroon, serum samples were collected from 395 healthy individuals from the BU endemic village of Mbandji 2. **B** In the Obom sub-district of the Ga-South district in Ghana, blood sera were collected from 96 BU patients (black) and 384 control individuals (grey) of the different age groups shown.

Demographic data as well as history of known previous mycobacterial infections were recorded for all participants at both sites. While the majority of individuals had no history of mycobacterial infections, eight study participants from Mbandji 2 reported to having had tuberculosis (2), leprosy (1) or BU (5). All control participants recruited in Ghana had no history of mycobacterial infection. The age distribution of study participants from Cameroon and Ghana is shown in Figure 1A and 1B, respectively.

Blood sera from the 875 individuals were tested for the presence of anti-18 kDa shsp antibodies in an ELISA format. In addition, 96 sera from children <5 living in Mbandji 2 were tested by Western Blot analysis for the presence of antibodies against this protein, as well as against a *Plasmodium falciparum* MSP-1 protein domain in order to assess the exposure and immune responses of child study participants to this mosquito transmitted parasite.

### ELISA

96-well Nunc-Immuno Maxisorp plates (Thermo Scientific) were coated with 0.25 µg recombinant *M. ulcerans* 18 kDa shsp per well in 100 µl phosphate-buffered saline (PBS) and incubated over night at 4°C. Plates were washed four times with washing buffer (dH_2_O, 2.5% Tween 20) before being incubated with blocking buffer 1 (5% skim milk in PBS) for 2 hours at room temperature (RT). After washing as described above, 50 µl of 1∶100 diluted human blood sera in blocking buffer 2 (1% skim milk in PBS) was added to each well and incubated for 2 hours at RT. Following a further washing step, 50 µl of 1∶8000 diluted goat anti-human IgG (γ-chain specific) antibodies coupled to horseradish peroxidase (HRP, SouthernBiotech) in blocking buffer 2 was added to each well and incubated for 1.5 hours at RT. Plates were washed and 50 µl TMB Microwell Peroxidase Substrate (KPL) was added per well. The reaction was stopped after 5 minutes using 0.16 M sulfuric acid. The absorbance was measured at 450 nm in a Tecan Sunrise microplate reader.

### Western blot analysis

15 µg of recombinant *M. ulcerans* 18 kDa shsp or 5 µg of a *Plasmodium falciparum* MSP-1 protein domain (amino acids 34-469 of strain K1) were separated on NuPAGE Novex 4–12% Bis-Tris ZOOM Gels with 1.0 mm IPG well (Invitrogen) using NuPAGE MES SDS Running Buffer (Invitrogen) under reducing conditions. After electrophoresis the proteins were transferred onto nitrocellulose membranes using an iBlot Gel Transfer Device (Invitrogen). Membranes were blocked with blocking buffer 3 (5% skim milk in PBS containing 0.1% Tween 20) and cut into thin strips. Membrane strips were then incubated with human blood sera at a 1∶1000 dilution in blocking buffer 3 for 2 hours at RT. Strips were repeatedly washed with 0.3 M PBS containing 1% Tween 20 and after that incubated with 1∶20'000 diluted goat anti-human IgG (γ-chain specific) antibodies coupled to HRP (SouthernBiotech) for 1 hour at RT. After a second washing step, bands were visualized by chemiluminescence using ECL Western Blotting substrate (Pierce).

### Data analysis

ELISA results were analyzed using GraphPad Prism version 6.0 (GraphPad Software, San Diego California USA) and R version 3.0.1 [15].

The distribution of antibody titres and the differences between two successive antibody titres are presented as box plots. These comprise a line for the median, edges for the 25th and 75th percentiles and traditional Tukey whiskers showing 1.5 times the interquartile distance. Dots on the graph represent individual points that lie outside that range.

We compared changes in OD between age categories in the Cameroon dataset using the Kruskal-Wallis test. Levene's test for homogeneity of variances was used to compare the degree of variation by age category. We compared the OD values for the Ghana matched cases and controls using conditional logistic regression.

The overall bias and variation between the first and second Ghanaian serum samples was estimated using the Bland-Altman method [16].

## Results

### Age distribution of BU incidence and *M. ulcerans* 18 kDa shsp specific serum IgG responses among individuals living in the Mapé Basin of Cameroon

**Figure 2:**
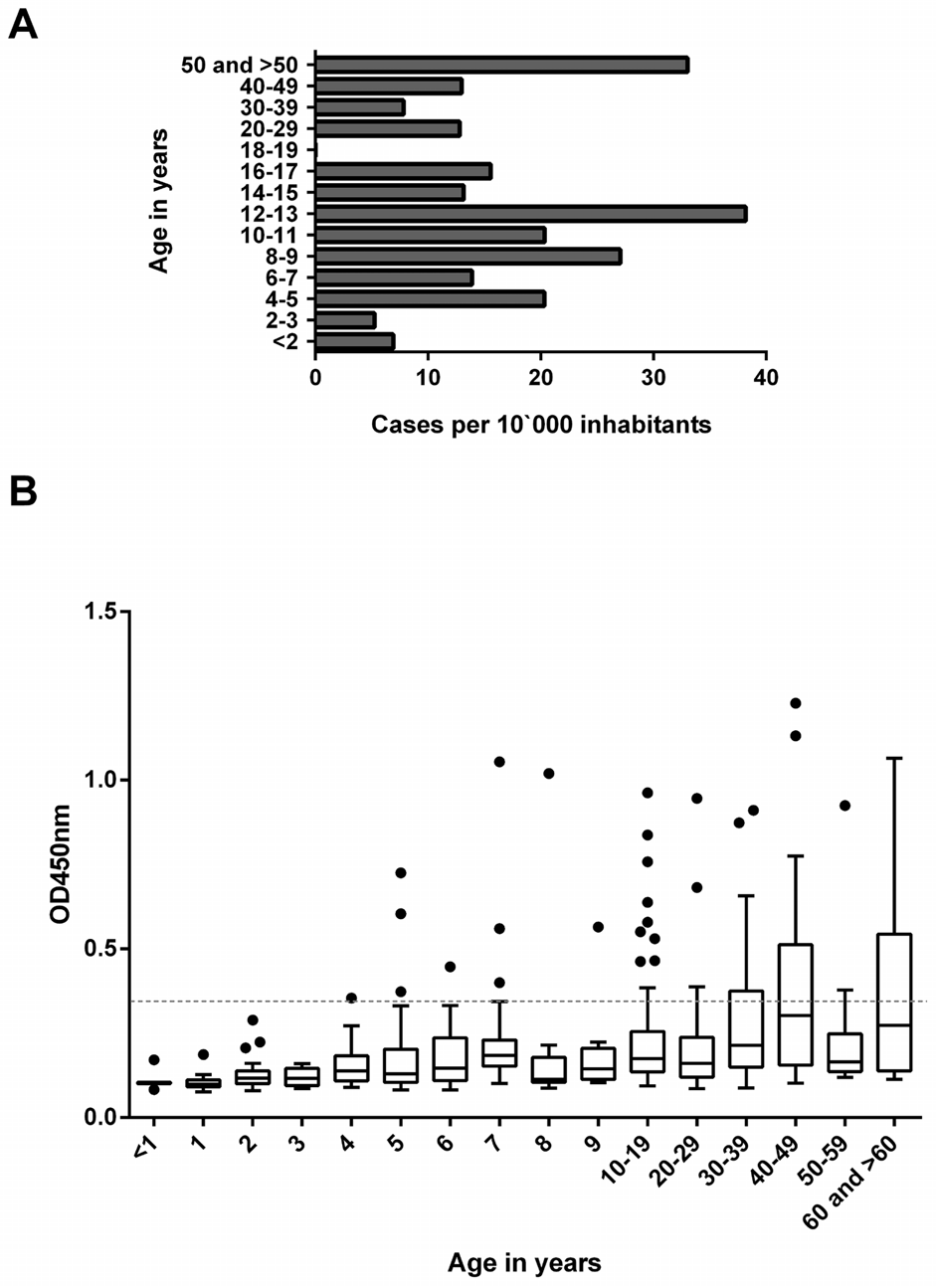
Age distribution of BU incidence and anti-18 kDa shsp IgG serum titres among healthy inhabitants of Mbandji 2. **A** Incidence of reported BU by age in the Bankim Health District (March 2010 – May 2013). **B** Boxplot of OD values of 1∶100 diluted serum samples from inhabitants of Mbandji 2 tested in an anti-*M. ulcerans* 18 kDa shsp IgG specific ELISA by age group. No IgG titres above the background level were observed for children below the age of four. The background response (OD<0.35) is indicated as a dotted line.

The age-specific BU incidence rates for the population in the Mapé Basin were calculated using 76 BU cases identified between March 2010 and May 2013. Based on these cases, a low incidence rate of BU was detected for children less than 4 years of age (Figure 2A).

**Figure 3:**
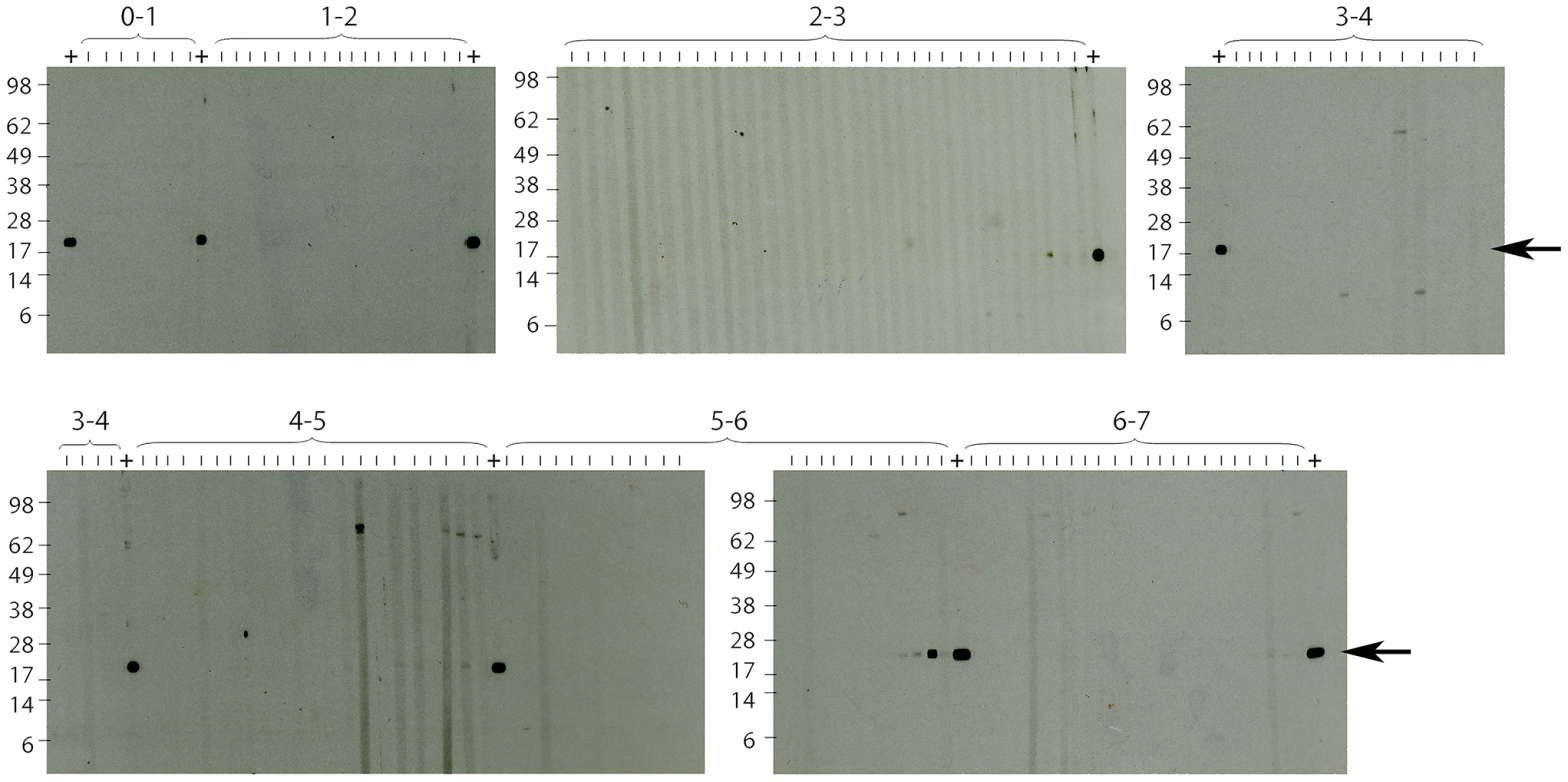
Western blot analysis of anti-18 kDa shsp IgG responses in children. Sera collected from children living in Mbandji 2 were tested for the presence of anti-*M. ulcerans* 18 kDa shsp IgG by Western Blot analysis. No specific bands were detected for very young children below the age of four. An ELISA positive control serum (OD = 0.963) was included between each of the age groups tested (+; arrow at band corresponding to size of the 18 kDa shsp).

The age-distribution of IgG titres against the *M. ulcerans* 18 kDa shsp for a cross-sectional survey of 395 individuals from the village Mbandji 2 is shown in Figure 2B. While high antibody titres were detected in individuals of all age groups over 4 years, none of the children younger than 4 years showed an ELISA IgG titre above the background, which was determined by Western Blot analysis as OD < 0.35. Analysis of the sera sampled from children less than 7 years old by Western Blot analysis showed no specific bands representing IgG antibodies against the 18 kDa shsp for sera from children <4 years of age (Figure 3). In contrast, Western Blot positive sera were found in all tested age groups >4 years old. Since very weak IgG titres were recorded for some of the sera from 4 year olds, sero-conversion may start in some children around this age.

### Serum IgG responses against a domain of *Plasmodium falciparum* merozoite surface protein 1 among children living in the Mapé Basin of Cameroon

IgG titres against a recombinant fragment of MSP-1 were determined by Western Blot analysis.

**Figure 4:**
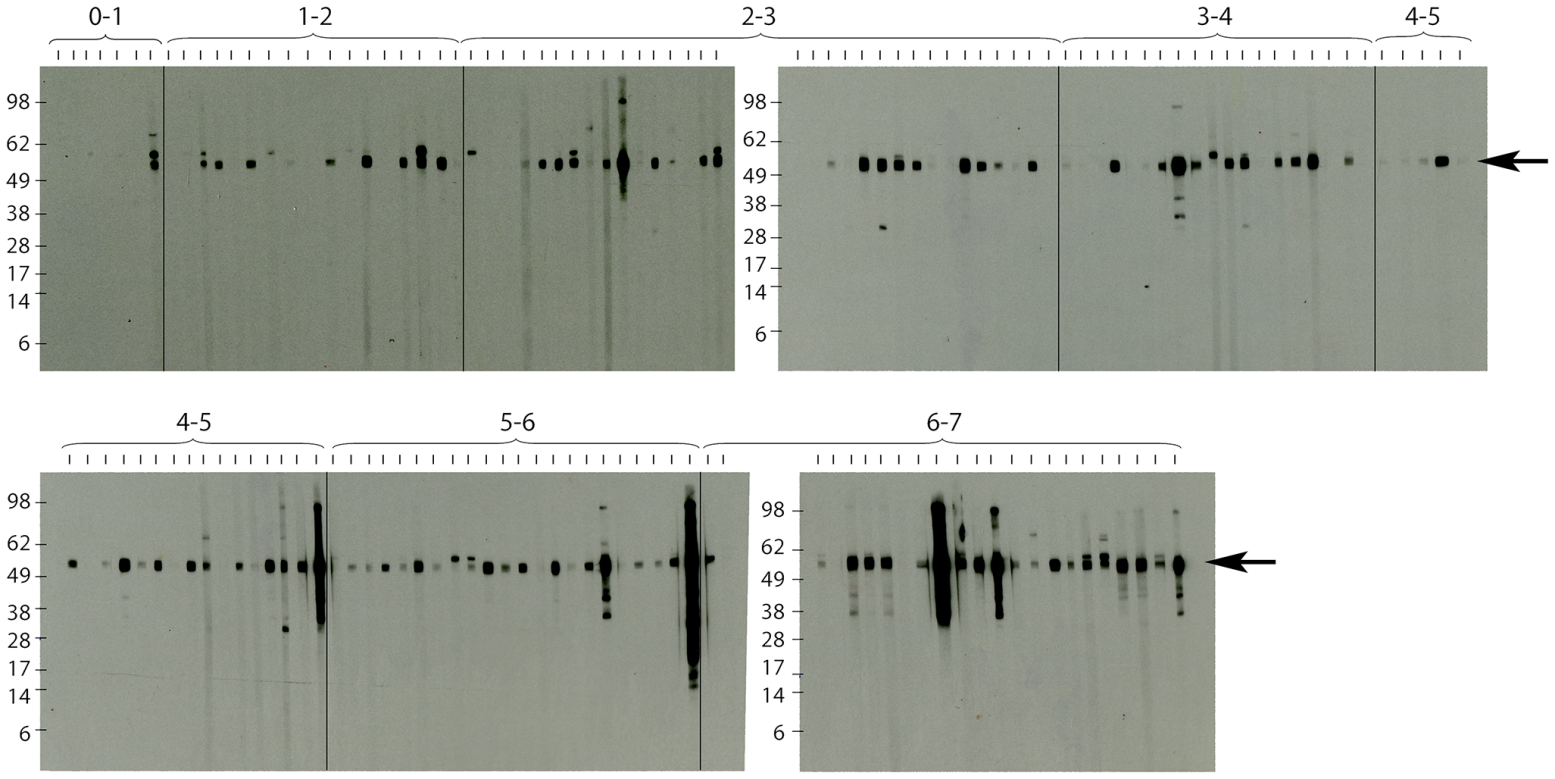
Western blot analysis of anti-*P. falciparum* MSP-1 IgG responses in children. Sera from children living in Mbandji 2 were tested for IgG responses against a domain of the *P. falciparum* MSP-1 protein by Western Blot analysis. Specific bands were detected in the majority of individuals of all tested age groups above 1 year. The band corresponding to the specific signal of the MSP-1 protein domain is indicated with an arrow.

In contrast to the lack of antibody responses against the 18 kDa shsp in children <4 years old, serum IgG responses against a *P. falciparum* malaria parasite MSP-1 domain were detected in all age groups tested. Strong staining of the MSP-1 band was observed for the majority of sera collected from children between one and seven years of age as well as for one of the infants (Figure 4).

### Stability of anti-18 kDa shsp IgG titres

**Figure 5:**
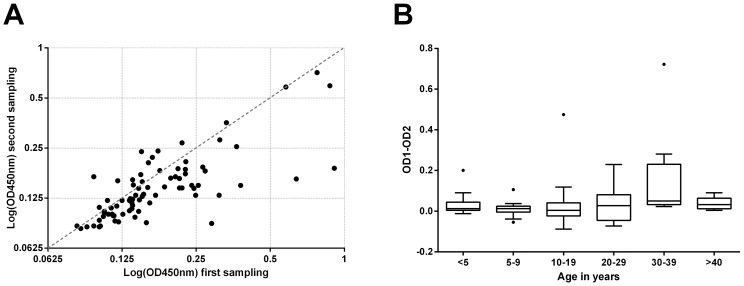
Changes in anti-*M. ulcerans* 18 kDa shsp IgG titres in sequentially collected serum samples. **A** IgG titres against the *M. ulcerans* 18 kDa shsp were determined in serial serum samples collected from 80 individuals. The majority of changes were small and most individuals showed a slightly decreased titre after one year. **B** Boxplot of differences in OD values between the two samples are shown by age group. Changes in antibody titres were most pronounced in young adults.

One year after the first serum collection in Mbandji 2, 80 of the 395 study participants were re-sampled. While only minimal changes in antibody titres against the 18 kDa shsp were recorded overall, more individuals had a decreased than an increased serum IgG level after one year (Figure 5A).

Increases in OD tended to be small and confined to the older children and young adults (Figure 5B). The most distinct changes, characterized by a marked decrease of antibody titres between the two surveys, occurred in young adults.

There was a significant association between age group and the absolute change in OD (Kruskal-Wallis test p = 0.01) and borderline evidence of an association between the variation in changes in OD and age group (Levene's test for homogeneity of variances, p = 0.08).

### *M. ulcerans* 18 kDa shsp specific serum IgG responses in BU patients and control individuals living in the BU endemic Densu River Valley of Ghana

*M. ulcerans* 18 kDa shsp specific IgG titres were also determined in sera from 96 BU patients and 384 healthy matched control individuals living in a second BU endemic site in West Africa, the Densu River Valley in Ghana. Each serum sample was tested twice, once in each of two independent experiments (Figure S1). Negligible overall bias between experiments was observed with the mean difference (OD1-OD2) of 0.024. There was also a reasonably small variation in the individual differences with the 95% limits of agreement from −0.0796 to 0.1278.

**Figure 6:**
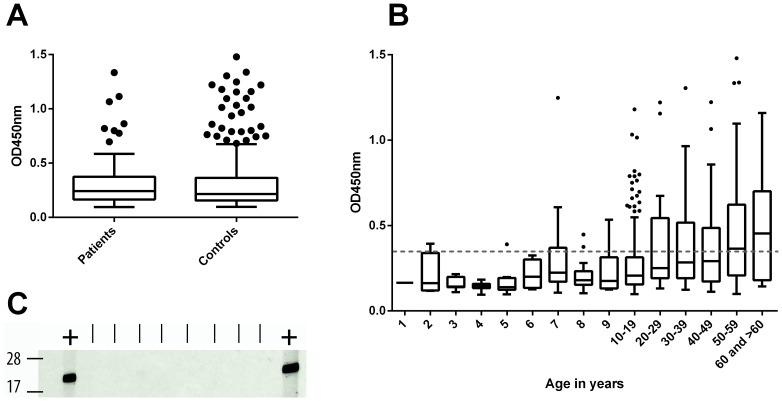
Anti-*M. ulcerans* 18 kDa shsp IgG titres in sera from Ghanaian BU patients and control individuals. **A** Box plots showing OD values of 1∶100 diluted sera from Ghanaian BU patients (n = 96) and control individuals (n = 384) tested in an anti-*M. ulcerans* 18 kDa shsp IgG specific ELISA. **B** Boxplot showing the distribution of OD values for BU patients and control individuals for different age groups. No IgG titres above the background level were observed for children below the age of 5 years. The background response (OD<0.35) is indicated as a dotted line. **C** Sera from eight 2-year-old children were tested by Western Blot analysis to reconfirm the absence of anti-*M. ulcerans* 18 kDa shsp IgG. An ELISA positive control serum (OD = 0.76) was included (+) flanking the tested sera.

There was no evidence of a difference in the ELISA OD values between the cases and controls (p = 0.99) (Figure 6A).

While sero-responders were identified in all age groups of individuals more than 6 years old, none of the sera from children younger than 5 years exhibited a distinct anti-18 kDa shsp IgG titre (Figure 6B). Western Blot analysis of sera from 2-year-old children confirmed the absence of anti-*M. ulcerans* 18 kDa shsp IgG in these samples (Figure 6C). Results of representative subsets of sera which tested negative, moderately positive or highly positive by ELISA were reconfirmed by Western Blot analysis, showing good agreement between ELISA OD values and Western Blot band intensities (Figure S2).

## Discussion

A high degree of antigenic cross-reactivity among mycobacterial species complicates investigations on *M. ulcerans*-specific humoral immune responses. However, the immunodominant 18 kDa shsp [17], which is overexpressed in *M. ulcerans* [18], represents a suitable serological marker for exposure to *M. ulcerans* [14]. Diverse outcomes of infection with other mycobacteria, such as *M. tuberculosis* and *M. leprae* have been associated with both host and pathogen factors. While only one study has investigated a possible association between BU and host genetics [19], various behavioural factors that may lead to increased risk to develop the disease have been reported, with poor wound care, failure to wear protective clothing, and living or working near water bodies being the most common risk factors identified [20]. While the generalization persists that children <15 years old are most affected by the disease [13], our recent survey for BU in the Mapé Basin [11] and continued monitoring of new BU cases in this region have revealed that the risk of BU is as high in individuals above the age of 50 as in young teenagers and that very young children below the age of four are underrepresented among cases when adjusting for the population age distribution. Data of our previous sero-epidemiological investigations revealed that the proportion of individuals from a BU endemic area showing serum IgG titres against the 18 kDa shsp of *M. ulcerans* is comparable for all age groups >5 years [14]. Results of the present study, including for the first time a substantial number of serum samples from children <5 years of age, showed that children of this age group have not yet sero-converted. Hence, young children appear to be considerably less exposed to *M. ulcerans*. This reduced exposure may be explained by the smaller movement radius away from the house of these very young children. Although, these small children do leave the house, they usually do so being carried by a caregiver and are therefore not in direct contact with the environment, at more distant places from their homes. No significant difference could be observed when comparing anti-*M. ulcerans* 18 kDa shsp antibody titres between BU patients and controls. This may be related to the immune-suppressive effect of mycolactone and concurs with the lack of a serological response in experimentally infected mice (unpublished data).

The results of a case-control study carried out in a BU endemic region of south-eastern Australia indicated reduced odds of having BU for individuals who frequently used insect repellent and increased odds for those who were bitten by mosquitoes [21]. In African BU endemic settings, the highly focal transmission of *M. ulcerans* haplotypes [12], [22], [23], as well as the distribution pattern of BU lesions on the body [11], speak against an exclusive role of mosquito vectors in transmission. Here we observed in children <5 years frequent sero-conversion for the MSP-1 antigen of the mosquito-transmitted malaria parasites in the absence of an IgG response against the *M. ulcerans* 18 kDa shsp. The age distribution of BU cases and the relatively abrupt changes in this risk of contracting BU with age do not speak for transmission of BU by mosquito species commonly found within the small movement radius of very young children.

Within the framework of our analyses, blood was collected for a second time from a limited number of participants one year after the first sample. Results of this pilot study showed that anti-18 kDa shsp IgG titres were relatively stable in older adults. Future studies of the age-related changes in behaviour of three to six year old children, monitoring of their movement radius and water contact patterns in combination with larger longitudinal serological and environmental studies may have the potential to shed further light onto the mode of transmission and relevant environmental reservoirs of *M. ulcerans*.

## Supporting Information

Figure S1 **Duplicate ELISA testing of sera.** All serum samples collected from individuals living in the BU endemic Densu River Basin of Ghana were tested twice (screen 1 and screen 2).
(TIF)

Figure S2 **Reconfirmatory Western blot of randomly chosen Ghanaian sera.** A subset of sera from Ghana which tested moderately positive (OD = 0.58–0.64), negative (OD = 0.1) and highly positive (OD = 1.0–1.2) by ELISA were tested by Western Blot analysis. Specific bands were detected for ELISA positive sera, while no signal was obtained for ELISA negative sera.
(TIF)
