# Supplementary figures and images for: Late Onset of the Serological Response against the 18 kDa Small Heat Shock Protein of Mycobacterium ulcerans in Children
Source: PLoS Negl Trop Dis. 2014 May 22;8(5):e2904. doi: 10.1371/journal.pntd.0002904 (PMC4031220; doi:10.1371/journal.pntd.0002904)

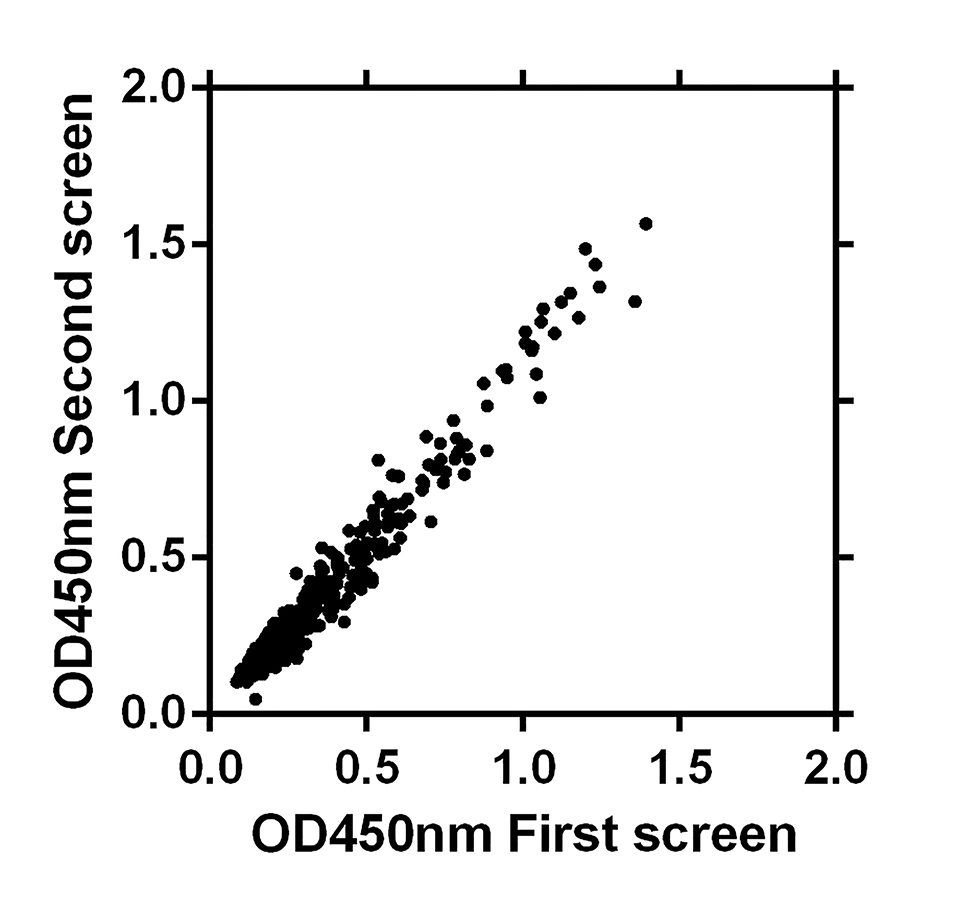

Supplement: Figure S1 — Duplicate ELISA testing of sera. All serum samples collected from individuals living in the BU endemic Densu River Basin of Ghana were tested twice (screen 1 and screen 2). (TIF) [file pntd.0002904.s001.tif]

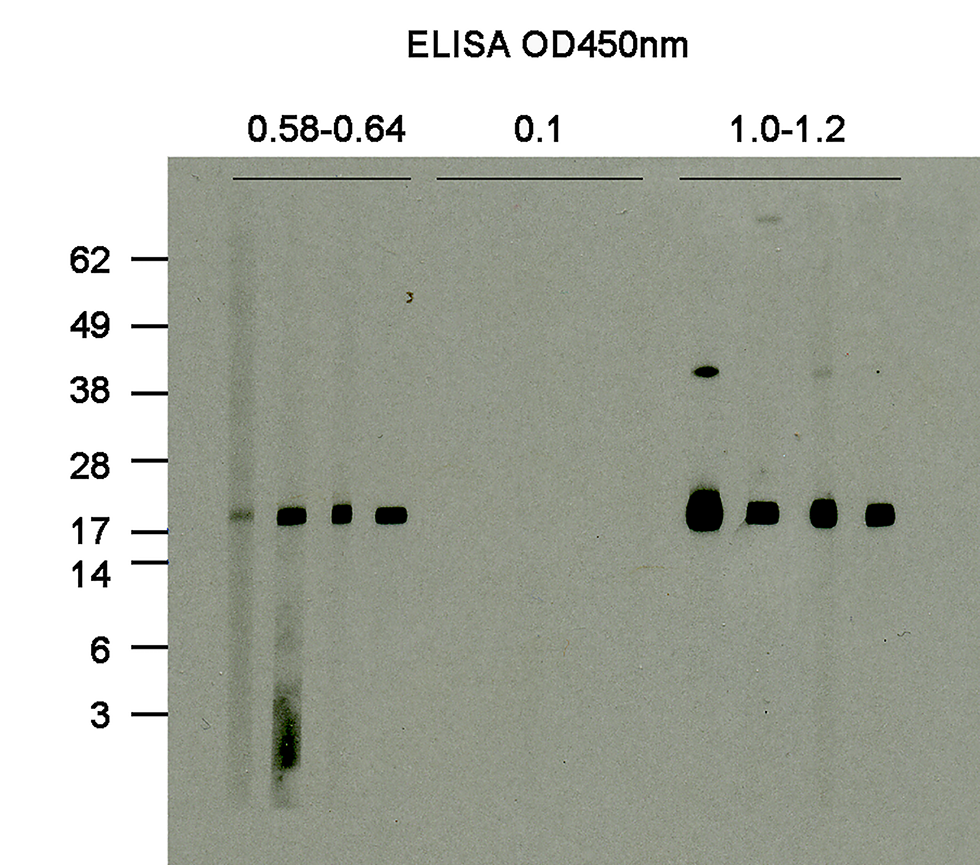

Supplement: Figure S2 — Reconfirmatory Western blot of randomly chosen Ghanaian sera. A subset of sera from Ghana which tested moderately positive (OD = 0.58–0.64), negative (OD = 0.1) and highly positive (OD = 1.0–1.2) by ELISA were tested by Western Blot analysis. Specific bands were detected for ELISA positive sera, while no signal was obtained for ELISA negative sera. (TIF) [file pntd.0002904.s002.tif]
